# Editorial: A year in review: discussion in molecular innate immunity

**DOI:** 10.3389/fimmu.2023.1255050

**Published:** 2023-08-08

**Authors:** Francesca Granucci

**Affiliations:** Department of Biotechnology and Biosciences, University of Milano-Bicocca, Milan, Italy

**Keywords:** innate immunity, innate immune receptors, fibroblasts, cGAS-STING, neutrophils, COVID-19

Molecular innate immunity refers to the early defense mechanisms that organisms possess against pathogens, which are primarily mediated by innate immune cells, their recognition receptors and mechanisms of defense. The understanding of this intricate system has significant implications for both basic science and clinical applications.

The field of Molecular Innate Immunity is a rapidly evolving research area, with new discoveries and advancements constantly emerging. In this Research Topic are a few areas that have attracted attention summarized in five reviews and two original articles.

Cavagnero and Gallo in their review aim to consolidate current literature on the innate immune functions of fibroblasts in barrier tissues, shedding light on the previously overlooked significance of these cells in immunity. While fibroblasts have traditionally been recognized as structural cells involved in tissue and scar formation, recent research has revealed their remarkable their remarkable heterogeneity and active participation in immune defense. Specifically, studies investigating barrier tissues like the skin, gut, and lung have demonstrated that certain fibroblasts possess the ability to directly detect pathogens and danger signals. These immune-responsive fibroblasts contribute to host defense by engaging in antimicrobial activity, recruiting immune cells, and producing inflammation-related cytokines and lipid mediators.

Song et al. provide insight into the immune response, the regulation of cGAS in the nucleus, and the potential mechanisms that prevent self-DA recognition through post-translational modifications. Innate immunity acts as the primary defense against microbial and viral attacks by establishing a protective barrier. Cytoplasmic pattern recognition receptors play crucial role in sensing foreign or host-origin pathogens, triggering immune responses. The cGAS-STING pathway is a major pathway that respond to microbial DNA, DNA viruses, and self-DNA derived from genome instability or mitochondria. While cGAS was initially thought to function in the cytoplasm, recent evidence suggests its presence in the nucleus, where it is involved in DNA damage repair. In this context, understanding the balance of cGAS function in the nucleus and cytoplasm, as well as its avoidance of recognizing host DNA, is crucial.

In another review on innate immune receptors Almeida-da-Silva et al. highlight that the field of NOD-like receptors (NLRs) has expanded in the last two decades, uncovering ligands and conditions that activate NLRs and the consequences of their activation in cells and organisms. NLRs paly crucial roles in various functions, including MHC molecules transcription and inflammation initiation. While some NLRs are directly activated by ligands, others may have indirect effects. The authors emphasize that ongoing research will further elucidate the molecular mechanisms of NLR activation and their physiological and immunological implications.

Krzyzanowski et al. address the important topic of inherited defect in neutrophil number or function. They concentrate on how these defects can lead to autoimmune and autoinflammatory phenomena, starting from the assumption that infectious complications in these disorders are generally well understood. In this review, they explore the clinical impact of autoimmunity and autoinflammation in individuals with neutrophil defects, seeking common patterns and delving into potential mechanisms and emerging treatment approaches. By examining this interplay, they aim to shed light on the complex relationship between neutrophil disorders and autoimmune/autoinflammatory manifestations, paving the way for improved understanding and management of these conditions.

This Research Topic contains also a review on COVID-19. Nilsson et al. focus on the impact of COVID-19 on the immune system. They start from their recent study in Frontiers of Immunology revealing that the intravascular innate immune system (IIIS) is highly activated in severe COVID-19 cases with acute respiratory distressed syndrome (ARDS), suggesting its role in driving disease severity. In this review they elucidate not only the physiological function of the IIIS but also highlight its strong proinflammatory effects observed in COVID-19 and other pathological conditions such as ischemia-reperfusion injury and treatment involving direct contact between biomaterials and blood. The authors highlight how by exploring IIIS, the study sheds light on its crucial role in the acute innate immune response and its implications in various medical conditions and treatments.

Finally, the two original articles concentrate one on the role of CD13 on neutrophils and the second one on the potential negative effect of polyps in the production of type I IFNs during Rhinovirus infection.

In particular, Pérez-Figueroa et al. focus on Aminopeptidase N (also known as CD13), an ectopeptidase found on the cell membrane of myeloid cells. While CD13’s enzymatic activity regulates various bioactive peptides, recent studies have uncovered its ability to activate signal transduction pathways and mediate effector functions in monocytes and macrophages, including phagocytosis and cytokine secretion. Although CD13 is expressed in neutrophils, its role in these cells remains unclear. In this study the authors demonstrate that CD13 in human neutrophils can mediate phagocytosis through a signaling pathway involving Syk and PI3-K. CD13-mediated phagocytosis is associated with reactive oxygen species (ROS) production, and induces the release of neutrophil extracellular (NETs) and cytokine secretion. These findings suggest that CD13 acts as a membrane receptor capable of activating effector functions in human neutrophils.

Lee et al. address the problem of dysregulated immune responses to rhinoviral infection in chronic rhinosinusitis (CRS). It remains unclear whether inflammatory epithelial cells in CRS patients with nasal polyps exhibits deficiencies in producing antiviral interferons upon rhinovirus infection. This study aimed to compare the replication rates of rhinovirus 16 (RV16) and secretion of interferons, as well as the expression levels of pattern recognition receptors, in normal and inflammatory epithelial cells. The findings indicate that RV16 replication rates and the production of antiviral interferons were similar in both cell types, suggesting that the inflammatory epithelial cells of CRS patient with nasal polyps do not exhibit deficient or delayed antiviral interferon responses to RV16 infection.

The field of Molecular Innate Immunity is continuously evolving. With this platform Frontiers in Immunology helps staying up to date with the most recent developments and perspectives on this subject.

## Author contributions

FG: Writing – original draft.

